# Empathic perspective taking promotes interpersonal coordination through music

**DOI:** 10.1038/s41598-019-48556-9

**Published:** 2019-08-22

**Authors:** Giacomo Novembre, Zoe Mitsopoulos, Peter E. Keller

**Affiliations:** 1 University College London, Department of Neuroscience, Physiology and Pharmacology, London, UK; 2 Neuroscience and Behaviour Laboratory, Istituto Italiano di Tecnologia, Rome, Italy; 3 The MARCS Institute for Brain, Behaviour and Development, Western Sydney University, Sydney, Australia

**Keywords:** Human behaviour, Personality

## Abstract

Coordinated behavior promotes collaboration among humans. To shed light upon this relationship, we investigated whether and how interpersonal coordination is promoted by empathic perspective taking (EPT). In a joint music-making task, pairs of participants rotated electronic music-boxes, producing two streams of musical sounds that were meant to be played synchronously. Participants – who were not musically trained – were assigned to high and low EPT groups based on pre-experimental assessments using a standardized personality questionnaire. Results indicated that high EPT pairs were generally more accurate in synchronizing their actions. When instructed to lead the interaction, high and low EPT leaders were equally cooperative with followers, making their performance tempo more regular, presumably in order to increase their predictability and help followers to synchronize. Crucially, however, high EPT followers were better able to use this information to predict leaders’ behavior and thus improve interpersonal synchronization. Thus, empathic perspective taking promotes interpersonal coordination by enhancing accuracy in predicting others’ behavior while leaving the aptitude for cooperation unaltered. We argue that such predictive capacity relies on a sensorimotor mechanism responsible for simulating others’ actions in an anticipatory manner, leading to behavioral advantages that may impact social cognition on a broad scale.

## Introduction

Synchronous behavior is a universal means of communication and cooperation observable in diverse species^1–4^. In humans, this kind of behavior can be witnessed during activities such as audiences clapping their hands in unison, military units marching, or groups of athletes or dancers coordinating their movements in space and time^5–9^. Joint music making is a pervasive example of this phenomenon^10^. Even a group of perfect strangers might end up singing together, for example during a competitive sporting game or a religious ritual – more likely so if the individuals support the same team, or believe in the same god, of course.

When multiple individuals coordinate their movements in order to achieve interpersonal synchrony, the group benefits in terms of enhanced interpersonal bonding and social cohesion^11,12^. Such pro-social effects of interpersonal synchronization have been reported in numerous laboratory studies using a variety of tasks^13–23^. For instance, dyadic finger tapping in synchrony is more likely to increase feelings of affiliation^18^, trust, and likeability^16,24^. These effects appear early in ontogenetic development, as evidenced by the finding that 14-month old infants, if moved in synchrony with another individual by an experimenter, are subsequently more likely to engage in cooperative helping behavior with that individual^19,25^. Even the mere observation of synchronous group behavior can augment perceived social cohesion^26,27^.

These previous studies demonstrated that interpersonal synchrony is a sufficient condition to boost interpersonal pro-social processes. However, this is only one part of the story. Complementarily to these observations, some studies have shown that social personality traits can also affect interpersonal coordination skills. For instance, in one study, it was reported that individuals affected by Social Anxiety Disorder are impaired in leading a task requiring interpersonal coordination^28^. Another study showed that individuals who have high internal Locus of Control (according to a questionnaire assessing to what extent individuals assume life events to be contingent on their personal behavior) are relatively good leaders in the sense that they prioritize stabilizing their own action timing over synchronization with an unreliable virtual partner^29^. Taken alongside the above evidence, these results suggest that the link between synchrony and prosociality might be bidirectional: increased interpersonal coordination can boost social behavior, but also enhanced social behavior can boost interpersonal coordination skills^6^. Yet, the sensorimotor and cognitive mechanisms mediating this bidirectional relationship remain mysterious, as do the personality traits associated with individual variations in the functioning of these putative mechanisms.

Here we hypothesized that a specific social personality trait that might influence interpersonal coordination behavior is empathy, which is generally defined as the human capacity to respond to – and share – experiences of others^30,31^. Psychological research indicates that human experiences can be shared with others at multiple levels, ranging from sharing others’ affective states to the cognitive ability to reason about another’s beliefs, thoughts, or intentions by taking their perspective^31–33^. Thus, empathy can be viewed as a multidimensional construct having distinct components^34–36^. The component that we investigated here is Empathic Perspective Taking (EPT), which can be assessed using standardized questionnaires^31,32^ that index how spontaneously one individual adopts others’ perspectives in everyday life situations. Thus, EPT will hereby be discussed as a proxy of empathic skills relevant to social cognition in general and interpersonal synchrony in particular.

Because interpersonal synchronization requires multiple individuals not only to be precise, but also to anticipate others’ behavior^6,37–40^, we hypothesized that more empathic individuals might perform relatively well on a task requiring interpersonal synchronization due to superior predictive skills. This hypothesis was motivated by a number of observations made in the field of cognitive neuroscience. Specifically, research using neuroimaging and brain stimulation techniques has revealed that more empathic individuals possess an enhanced “motor simulation” mechanism, which is capable of representing another’s actions in terms of the neural resources necessary to execute the same action^41–45^. Further research has shown that motor simulation has a predictive character, i.e. it supports the capacity to anticipate another’s upcoming actions in the observer’s brain^45–52^. Taken together, this body of research suggests that more empathic individuals may benefit from enhanced motor simulation, and thereby superior predictive skills when it comes to synchronizing their actions with the actions of others.

**Figure 1:**
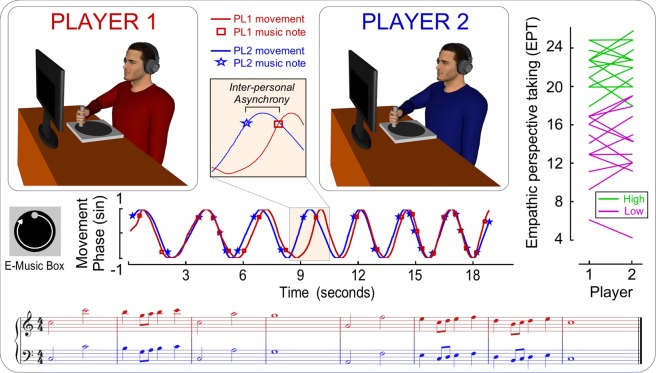
Experimental paradigm. Pairs of musically untrained participants use E-music boxes to perform a musical melody together. The E-music box transforms rotatory movements into a pre-programmed musical melody whose tempo varies according to the rotation velocity. When the E-music box is rotated at constant velocity, the melody is played with correct rhythm and constant tempo (for details see)^59^. Participants are instructed to synchronize their musical outputs as accurately as possible. Their movements are therefore highly controlled and entail continuous rotations of their right arms. The latency of movements and outputted musical tones are used to study interpersonal coordination accuracy, as well as leader-follower relations. Before taking part in the study, each participant is paired with another individual having a similar empathic perspective taking (EPT) score (i.e. their score difference is not larger than 3). Eventually, groups of relatively higher and lower EPT pairs are compared one another following a median split.

To test this hypothesis, we employed a task involving an ecologically valid form of interpersonal synchronization that could also be tightly controlled under laboratory conditions, i.e. a joint music making task^10^. Interpersonal coordination through music is a universal and natural form of social interaction, with widespread examples including ritual and congregational activities or infant-caregiver communication^53,54^. Musical coordination is often studied in expert musicians^10,55–58^, but it is also advantageous to do so in the general population by exploiting fundamental musical predispositions that have evolved in humans^59^. The latter approach is well suited to address our goal because it does not confound personality traits related to musicianship with empathic perspective taking. To this end, here we used a custom-built instrument that was developed to allow individuals without musical training to perform music collectively: the E-music box^59^. The E-music box transforms cyclical rotatory movements into a preprogrammed musical melody whose tempo varies according to the velocity of the rotation (Fig. 1). The tunes can be played with correct rhythm and constant tempo by rotating the handle with constant velocity.

We recruited a group of individuals who had never received formal musical training. We used a standardized questionnaire to assess each individual’s empathic score^32^, and then formed pairs of individuals who scored similarly on the Empathic Perspective Taking (EPT) subscale, indexing an individual’s spontaneous tendency to adopt the psychological point of view of others (ranging from 0 to 28). In order to minimize within-pair EPT score variability, participants were paired with partners whose EPT score were within 3 points on the scale (Fig. 1). We then invited these pairs into the lab, and asked them to perform the joint music making task as accurately as possible. To the extent that higher empathic skills are generally associated with higher interpersonal coordination skills, we predicted that those pairs formed by relatively more empathic participants would be more accurate in the interpersonal coordination task. To address this, we computed the average asynchrony between musical tones from the two parts that were meant to be synchronous and compared it across two groups of pairs scoring relatively higher or lower on the EPT assessment (following median split).

Furthermore, to test the hypothesis that EPT facilitates interpersonal coordination by affecting temporal prediction, we manipulated leader-follower relations via instructions. Specifically, the paired participants were asked to perform the joint music making task across three conditions: one where one participant was instructed to lead and the other to follow, a second condition where these roles switched, and finally a condition during which no leadership was assigned. The assumption behind this manipulation was that prediction demands vary with leadership role: Leaders are primarily responsible for controlling their own actions^29^ while followers are required to attend to the leader in order to predict his or her action timing, and to adapt to interpersonal timing discrepancies^59–61^. It is therefore optimal for leaders to make their actions as predictable as possible, a strategy that has been previously observed in a variety of joint action contexts^62,63^ and is referred to as a “coordination smoother”^64^. This strategy should facilitate interpersonal coordination accuracy and stability to the extent that the follower can make use of the leader’s timing cues. Thus, if EPT increases the use of such cues, then high EPT followers should be better able to predict, and therefore temporally align, their actions with the leaders’ actions.

## Materials and Methods

### Participants

We recruited 58 healthy individuals (39 female, age mean ± std = 25.64 ± 8.54 years). All participants were categorized as non-musicians based on a screening questionnaire (the Ollen Musical Sophistication Index, OMSI^65^). All participants reported having normal hearing. All procedures were in accordance with the Declaration of Helsinki and were approved by the human research ethics committee of Western Sydney University (approval number: H10487). Informed consent was obtained from all participants (each being at least 18 years old when the experiment was conducted).

### Pre experiment empathy-based assessment

Before taking part in the experiment, empathy was assessed in all participants using the Interpersonal Reactivity Index (IRI)^31,32^, a questionnaire that was administered online. The IRI consists of 28 self-report items measuring empathy as a multidimensional construct. The IRI distinguishes between affective and cognitive empathy as distinct components of the construct. The subscale of interest here was perspective taking (PT), an aspect of cognitive empathy, which refers to one’s tendency to adopt another’s viewpoint. The PT score ranges between 0 (lowest perspective taking tendency) to a maximum of 28 (highest perspective taking tendency). Following the collection of the above-specified empathic PT scores (EPT), we paired together participants having similar EPT scores (i.e. an absolute score difference not larger than three).

Next, we performed a median split on the sample according to the participants’ EPT score (averaged within each pair). This resulted in two groups that are hereafter referred to as Low EPT (n = 13 dyads; 1 male-male, 6 female-female, and 6 mixed dyads) and High EPT (n = 13 dyads; 2 male-male, 5 female-female, and 6 mixed dyads) groups. Sex ratios were therefore comparable across groups. Yet, we acknowledge that, despite sex being similarly distributed across EPT groups, the sample included more females than males. It would be useful to control this more strictly in future investigations, given that sex and gender can affect both empathy and interpersonal coordination^66,67^.

Three additional pairs falling on the median were excluded in order to form two clearly distinguishable groups. The mean EPT score of each group was 13.73 ± 3.53 for the Low EPT group and 22.35 ± 1.93 for the High PT group. None of the paired participants were friends or romantically involved prior to taking part in the experiment. Low and High EPT groups were comparable in terms of music and dance experience, as assessed using the OMSI scores (t < 2, p > 0.05). Furthermore, the percentage of participants who were born and raised in Australia (16 out of 26 [High EPT] and 15 out of 26 [EPT group]), and the level of education (rated on a 1–8 scale ranging from school certificate to doctoral degree: 3.62 ± 1.49 [High EPT], 3.85 ± 1.75 [Low EPT]), were comparable across groups.

### Apparatus and musical material

Participants could control the timing of a pre-programmed melody using the E-Music Box^59^. The E-Music Box is a small disk jockey’s turntable with an exterior handle attached, which enables the participant to rotate the turntable in order to produce the pre-programmed melody (Fig. 1; for technical details see^59^). The melody consisted of the first eight bars from the tune *Somewhere over the Rainbow*, originally written for the 1939 movie *The Wizard of Oz*. This tune was chosen for its widespread popularity and familiarity, which was assumed to facilitate the task for the musically untrained participants. Importantly, all participants reported being familiar with this melody and, when asked to rate familiarity on a 1–5 Likert scale, the two EPT groups yielded comparable scores (High EPT: 3.77 ± 1.42; Low EPT: 3.46 ± 1.39).

The score of the section of the tune used in the current study is presented in Fig. 1. The part higher in pitch (upper line in the score) and the part lower in pitch (lower line) were separated by two octaves (i.e. the fundamental frequency of the high pitch tones was four times greater than the fundamental frequency of the low pitch tones). The tone and register for the initial notes were C5 (higher pitch part) and C3 (lower pitch part). Musical Instrument Digital Interface (MIDI) files were created for each note in the score (using the software Max/MSP, with piano as the musical instrument). Each note had equal duration (500 msec) and loudness (which was controlled by applying a constant MIDI velocity of 120).

The outputs from the two E-Music Boxes were fed into a high-performance Dell computer (Dell OptiPlex 960, with dual 3.0 GHz Xenon processors). The computer was programmed to play an eighth note (quaver; i.e. half a beat) following a 45° rotation of the E-Music Box handle. A quarter note (crotchet; i.e. one beat) or a half note (minim; i.e. two beats) were played following 90° or 180° rotations. Hence, as long as the E-Music Box was rotated at constant velocity, the produced music would have the correct rhythmic structure.

### Procedure

Participants in the high EPT group and the low EPT group were invited to the lab in pairs and each individual was placed in one of two separate soundproofed rooms. Each room was equipped with a computer monitor (providing instructions to the participants), an E-Music Box and a pair of headphones (providing auditory feedback of their own and their partner’s performance). Participants were randomly assigned to play either the low-pitch or the high-pitch musical part.

A trial was structured as follows: First, a fixation cross was presented on the monitor (500 ms). This was followed by a curved arrow (3 s) indicating whether the E-Music Box was supposed to be played through clockwise or anticlockwise rotation of the handle. Subsequently, a metronome was presented (four beats, 750 ms intervals) while a symbol depicting an ear appeared (accompanied by the text “listen”). This was intended to provide a temporal cue helping the participants to establish the correct tempo and to start in synchrony. When the metronome ceased, a “GO” sign appeared on the monitor, at which point participants were instructed to begin producing music through the E-Music Boxes (using the right hand) and synchronizing their music with the music played by the partner. To familiarize participants with the procedure, each pair performed a few training trials before starting the experiment until they felt clear about the task.

All pairs performed 64 trials, which were grouped into 8 blocks of 8 trials. Participants rotated the E-Music Box turntable in the same or in the opposite directions with equal probability. The assignment of directions was counterbalanced across participants. Leadership instructions were provided at the outset of each block, and remained unchanged for the whole block. These instructions were provided by presenting text on the monitor stating either: “you lead” and “you follow” (4 blocks, with alternated instructions across participants) or “No Leadership” (4 blocks, with the same instruction for both participants). The order of the blocks and the trials within each block was randomized.

Note that repeating the same leadership instruction throughout a given block was meant to induce a stronger and clearer spontaneous lead-lag relationship. This could also be achieved by forming asymmetric pairs from the beginning of the experiment, as done in other studies^68,69^. Such an approach, however, would have also required additional control measures to determine who (within a pair) should be selected to be the leader or the follower. Thus, for the sake of simplicity, we did not opt for such an alternative approach.

### Data analysis

#### Interpersonal synchrony

Synchronization accuracy was measured in terms of absolute asynchronies, here corresponding to the absolute difference between partners’ complementary tone onset times (in ms), averaged within each trial. Trials associated with outlying asynchronies, i.e. deviating >3 SDs from the pair’s mean asynchrony across all trials, were excluded from further analyses. These constituted 0.96% of all trials.

We also measured how synchronization accuracy changed throughout the course of the musical piece (Fig. 1). To do so, we extracted movement timing, which was indexed by 57 consecutive time points representing when each participant completed a 45° rotation of the E-music box. Note that this is the angle corresponding to the movement necessary to play an eighth-note (half the musical beat interval), which is the shortest note duration in the target melody. Thus, considering that the to-be-played score comprised 7 full bars plus one single note associated with the 8^th^ bar, the total number of these events was 7 (full bars) × 8 (number of 45° rotations associated with performance of one bar) + 1 (the single note associated with the 8^th^ bar) = 57. The absolute difference between partners’ complementary time points provided an index of moment-by-moment synchronization throughout the musical piece.

#### Individual tempo stability

We computed the variability of each individual’s performance tempo in each condition. It was assumed that, in the condition where leadership was assigned, leaders would be relatively more stable in their tempo while followers would be more variable due to adaptation (via temporal error correction) to the leader’s timing^29,70^. Tempo variability was computed separately for each participant by (1) extracting information about movement timing for consecutive 45° rotations of each participant’s E-music box (see above), (2) first-order differencing successive time points to yield series of 45° rotation time intervals, and (3) calculating the coefficient of variation (CV) for each participant’s performance in each trial by dividing the standard deviation of 45° rotation time intervals by the mean 45° rotation time intervals. When this index was associated with a trial during which no leader was assigned, the average between the two players’ CVs was taken.

As with the interpersonal synchrony measure, we also examined how individual tempo stability changed throughout the course of the musical piece. To do so, we conducted a windowed analysis for which the coefficient of variation was computed within a moving window comprising 8 consecutive events (equivalent to 4 beats, or 1 bar) and moving in steps of 1 event. Thus, this analysis yielded timeseries of 50 windowed CVs, with the first windowed CV centered in between the 4^th^ and 5^th^ 45° rotation intervals, and the final centered in between the 52^nd^ and 53^rd^ interval.

#### Temporal leader-follower relation

To test the hypothesis that empathic followers are better able to predict leaders’ performance timing, we computed a continuous index of temporal leader-follower relation in the conditions where one participant was instructed to serve as the leader. This was done by extracting high-resolution phase information indexing continuous movement, i.e. the position of the E-music box handle over time (one sample every 10 ms, i.e. 100 Hz, with phase angles flipped for anticlockwise rotations, so that they were comparable to clockwise rotations). Next, continuous relative phase was computed by subtracting the unwrapped continuous phase of the follower from the unwrapped continuous phase of the leader within each trial^59^. Negative relative phase values indicate that leaders were leading the follower’s movements. Values closer to zero suggest that followers’ prediction of leaders’ performance timing was more accurate. (Note that computing relative phase using the unwrapped phase signals is important in order to guard against effects of phase wrapping, which can produce artificially high measures of synchrony, for example when the performances of two players are separated by a lag of a full cycle. Thus, this measure does not range between 0° and 180°, but between 0° and 360°(one full cycle) * the number of cycles necessary to perform the whole piece).

#### Note density

In order to assist in interpreting results with respect to factors such as information availability, we computed a descriptive index of note density associated with the musical piece used here. As displayed in Fig. 1, the number of musical notes was not uniform throughout the musical piece. For instance, some bars like the 2^nd^, the 6^th^ and the 7^th^ conveyed a larger number of musical events (a mixture of eight and fourth notes) compared to others such as the 1^st^, the 3^rd^ and the 5^th^ (where only two half notes were presented), or the 4^th^ and the 8^th^ (where only one full note was presented).

This information is important to consider because it provides an estimate of the amount of information that the two players could exchange during a given interval of the musical piece. This will be used to interpret the timecourses of the statistics illustrated above. To compute note density, we created a 57-elements array – representing all possible note positions available within the duration of our musical piece – and filled it in with either zeros (if no event was associated with a given position) or ones (if one event was present). Next, we computed the sum of these events using a moving window comprising 8 consecutive events and moving in steps of 1 event.

#### Statistics

*Interpersonal synchronization accuracy* was averaged across trials, separately for each condition and pair. In the main analysis, these averages were submitted to a single 2 × 2 mixed design analysis of variance (ANOVA). This ANOVA included a between-subjects factor EMPATHY (low EPT and high EPT) and one within-subjects factor LEADERSHIP (with and without). In a second analysis, we conducted a series of such ANOVAs, one for each of the 57 consecutive time points indexing when each participant completed a 45° rotation of the E-music box. This analysis was intended to reveal the timecourse of the effects yielded by the main analysis, as a function of the musical piece. Because this second analysis involved multiple statistical tests, it was necessary to correct for false positives. This was done using a cluster-based permutation test broadly used for timeseries analyses in neurophysiology^71^. The test entailed the following steps. First, neighboring significant time points (*p* < 0.05) were clustered together, and their cluster significance was computed by summing the F values of all points belonging to each cluster. Next, to assess the significance of each cluster, we permuted the original data by shuffling pairs’ averages, i.e. randomly assigning pairs’ data to (1) either high or low EPT groups and, within pairs, to (2) leadership conditions. After each permutation (n = 1000 iterations), the clusters with largest significance values were saved. These values were then used to generate a random significance distribution (1000 values). This random distribution was used to define a threshold (*p* = 0.05) against which the clusters obtained from the original data were compared.

*Individual tempo stability* (variability of performance tempo indexed by CV of 45° rotations) data were analyzed in a mixed design ANOVA with the between-subjects factor EMPATHY (low EPT and high EPT) and within-subjects factor LEADERSHIP (leader, follower, no leader). Data were averaged across the two individuals within a pair for each level of the LEADRERSHIP factor. As for the previous measure, a series of ANOVAs were also conducted separately for each of the 50 consecutive CVs in order to reveal the timecourse of the effects yielded by the main analysis. The cluster-based permutation test described above was used again to identify significant clusters while correcting for multiple comparisons.

*The temporal leader-follower relation* index in the leadership assigned condition was averaged and compared between the high EPT group and the low EPT group using a two-sample t-test. Before entering the averages into the statistical test, we normalized these values by subtracting the averaged leader-follower relation index computed for the condition where no leadership was assigned.

Finally, in order to explore the relationship between the timecourse of the effects yielded by the ANOVAs and the structure of the musical piece, we correlated the timecourse of the F values resulting from the ANOVAs conducted for each timepoint with windowed note density. These Pearson’s correlations were conducted after transforming the F-values and note density timeseries into z-scores.

## Results

### Interpersonal synchronization accuracy

**Figure 2:**
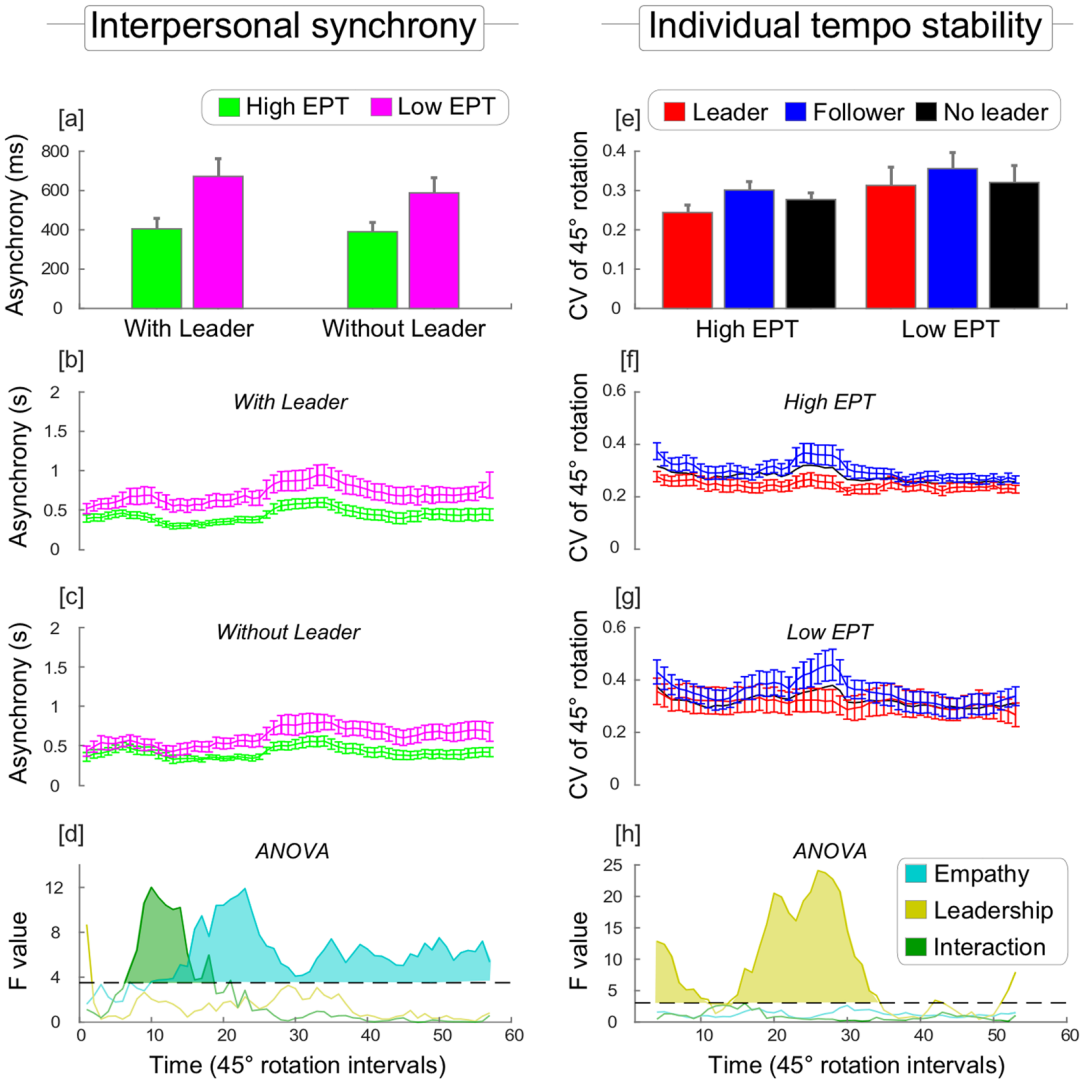
Interpersonal synchrony and individual tempo stability. Left (**a**–**d**): Interpersonal synchronization accuracy expressed in terms of asynchronies (i.e. the smaller the value, the higher the synchronization accuracy). Right (**e**–**h**): Individual tempo stability expressed in terms of the coefficient of variation of participants’ movements (i.e. the smaller the value, the more stable and predictable the performed tempo). The top row (**a**,**e**) displays measures averaged across all time points (each indexing 45° rotations of the E-music box). The second and the third rows display measures for each time point. The fourth row display series of F-statistic values obtained after running an ANOVA for each time point (the dashed line indexes significance threshold of each single test, while the shadowed areas indicate significance following correction for multiple comparison using the cluster-based permutation test). The results from the ANOVAs conducted on the averaged measures (displayed in the top row) are reported in the results section. Bars represent 1 standard error of the mean.

The ANOVA on mean absolute asynchrony data yielded a statistically significant main effect of EMPATHY, *F*(1, 24) = 6.76, *p* = 0.02. This indicated that the high EPT group yielded overall smaller asynchronies (mean ± SD; 397 ± 162 ms) compared to the low EPT group (630 ± 276 ms) (Fig. 2a). Thus, pairs composed of relatively more empathic individuals were more accurate in the interpersonal synchronization task. Both the main effect of LEADERSHIP, *F*(1, 24) = 1.13, *p* = 0.30, and the interaction between EMPATHY and LEADERSHIP were not significant, *F*(1, 24) = 1.02, *p* = 0.33. (An anonymous reviewer questioned the reliability of median-splitting the data because, in some circumstances, this can lead to artificial subgroups with no practical meaning. To address this issue, we also performed a Pearson correlation analysis between mean absolute asynchrony data from each pair and their EPT scores. The results of this analysis were in line with our conclusions: relatively lower asynchronies (i.e. higher synchronization accuracy) were associated with higher EPT scores (*r*(24) = −0.43; *p* = 0.02)).

**Figure 3:**
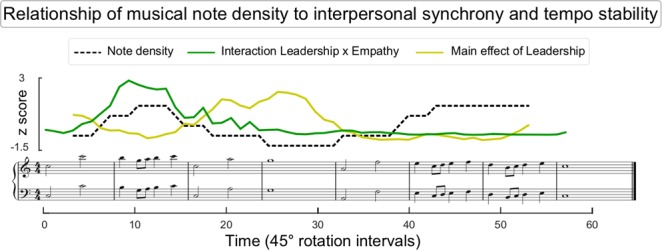
Relationship of musical note density to interpersonal synchrony and tempo stability. Estimated note density (dashed line) is plotted as a function of the musical score, with higher values representing higher density of notes within windows comprising 4 beats. The interaction between EMPATHY and LEADERSHIP associated with interpersonal synchrony (green line, Fig. 2) was stronger when note density was higher. However, the main effect of LEADERSHIP associated with individual tempo stability (yellow line, Fig. 2) was stronger when note density was lower. Statistics are reported in the results section. All timeseries were transformed into z-scores.

We next explored how these effects emerged as a function of timepoint during the musical piece. This analysis confirmed that high EPT pairs were generally more accurate than low EPT pairs (Fig. 2d; cluster-corrected significance: *p* = 0.01). Crucially, however, this analysis also indicated that when a leader was assigned, empathic pairs were relatively faster in establishing synchronization. As displayed in Fig. 2b,c, the enhanced accuracy associated to high EPT pairs had earlier onset when a leader was assigned. This was confirmed by a significant interaction between EMPATHY and LEADERSHIP, which was strongest for the timepoints associated with the second bar of the musical piece (Fig. 2d; cluster-corrected significance: *p* = 0.04). We noted that this bar corresponded to the first run of relatively short note durations, hence higher note density, suggesting that interactive benefits of empathy and leadership emerged when information about the partner’s timing started coming in at a relatively high rate. To confirm this suggestion, we ran a correlation between the timeseries of F values associated with the interaction between EMPATHY and LEADERSHIP (green line in Figs 2d and 3) and note density (Fig. 3). This analysis yielded evidence for a positive significant correlation *r*(48) = 0.27, *p* < 0.05.

In sum, high EPT pairs were more accurate in synchronizing than low EPT pairs. Furthermore, when a leader was assigned, the beneficial effect of empathy emerged earlier, presumably as soon as enough information about the partner’s timing was available.

### Individual tempo stability

Data for the variability of performance tempo (CV of 45° rotations) with and without leadership assignment are displayed in Fig. 2e. The ANOVA on these data revealed a statistically significant main effect of LEADERSHIP, *F*(2, 48) = 11.98, *p* < 0.001, indicating that variability of performance tempo was lower in leaders (0.283 ± 0.126) than followers (0.329 ± 0.118) when leadership was assigned, and intermediate when leadership was not assigned (0.304 ± 0.118). This result indicates that participants, when instructed to act as leaders, maintained more constant timing throughout the task^62,64,72^.

Although there is a numerical tendency for lower tempo variability in the high EPT (0.274 ± 0.065) than the low EPT individuals (0.329 ± 0.152), the main effect of EMPATHY was not significant, *F*(1, 24) = 1.32, *p* = 0.27. Likewise, while the effect of LEADERSHIP is numerically larger for the high EPT group than the low EPT group, the EMPATHY × LEADERSHIP interaction was not significant, *F*(2, 48) = 0.99, *p* = 0.38.

When we explored the timecourse of these effects as a function of the musical piece, it became apparent that the main effect of LEADERSHIP was driven by select sections of the musical piece, namely the 1^st^, the 3^rd^ and the 4^th^ musical bars (Figs 2f–h and 3). Consistent with this conclusion, the cluster-based permutation test identified two significant clusters. An earlier and relatively small one associated with the 1^st^ musical bar (Fig. 2h; cluster-corrected significance: *p* < 0.02), and a larger and later one associated with the 3^rd^ and the 4^th^ musical bars (Fig. 2h; cluster-corrected significance: *p* < 0.001). Looking at the musical score (Fig. 3), these bars contain a relatively low number of notes, and therefore were associated with sections of the musical piece during which less information about the partner’s timing was available. To test whether the main effect of LEADERSHIP was indeed stronger when less information about the partner’s timing was available, we ran a correlation between the timeseries of F values associated with the main effect of LEADERSHIP (yellow line in Figs 2h and 3) and windowed note density (Fig. 3). This analysis yielded evidence for a significant negative correlation, *r*(48) = −0.71, *p* < 0.001.

Thus, when participants were instructed to act as leaders, they maintained more constant timing, presumably in order to increase their predictability and help followers to synchronize^62,64,72^. This behavior was most evident during sections of the musical piece during which participants could exchange little information and, most importantly, was not different across high and low EPT groups.

### Temporal leader-follower relation

Having established that Leaders generally increased their temporal predictability, irrespective of their empathic score, we hypothesized that Followers might be better able to use this information to predict leaders’ movement timing and thus improve interpersonal synchronization. To test this hypothesis, we extracted movement information (i.e. phase, indexing movement of the music box with high temporal resolution) and computed a continuous index of relative phase in the conditions where one participant was instructed to serve as the leader.

**Figure 4:**
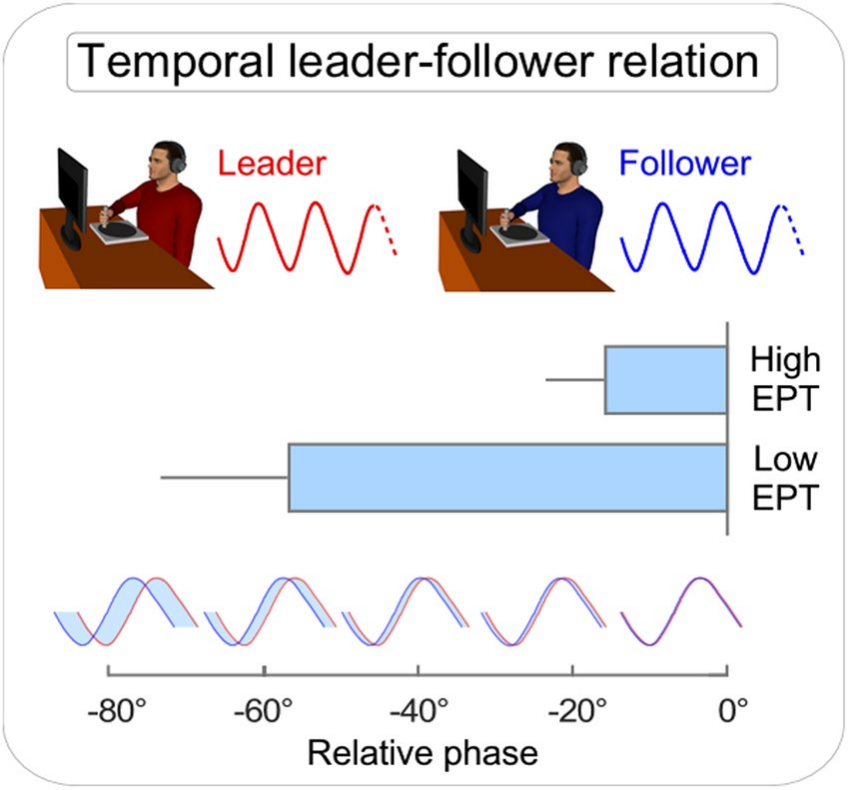
Temporal leader-follower relation. Movement information from both players was codified in terms of phase, indexing the (circular) rotation of the E-music box. Continuous relative phase was computed in order to quantify the extent to which followers’ movements lagged behind leaders’ movements when leadership roles were assigned. Compared to low EPT followers, high EPT followers lagged behind leaders to a smaller degree. Bars represent 1 standard error of the mean.

The mean relative phase for high EPT and low EPT groups is shown in Fig. 4. As should be expected, relative phase was generally negative, indicating that followers’ movements generally lagged behind leaders’^59,73^. Importantly, a statistically significant difference between relative phase in the high and the low EPT group indicated that this tendency was relatively smaller in the high EPT group, *t*(12) = −2.37, *p* = 0.03. Thus, high EPT followers lagged behind leaders to a smaller degree (−15.78° ± 29.11°) than low EPT followers (−56.85° ± 62.50°). This result is consistent with the hypothesis that high EPT pairs benefitted from superior predictive skills, which allowed followers to synchronize better with the leaders.

## Discussion

Here we compared interpersonal coordination skills across two groups of individuals that were relatively high or low in empathic perspective taking (EPT). We did so in the context of a musical joint action task that required musically untrained pairs of ‘non-musician’ participants to synchronize the outputs of two E-music boxes, i.e. electromechanical devices that transform rotatory movements into pre-programmed music whose tempo changes as a function of movement rotation speed (Fig. 1)^59^.

We made three primary observations. High EPT individuals were overall (1) more accurate at synchronizing with their partners, as compared to low EPT pairs (Fig. 2a). This result indicated that individuals who possess higher social skills – in this case empathic skills – have higher interpersonal synchronization skills. We also explored leader-follower relations. We observed that (2) when the participants were instructed to act as leaders, they maintained steadier timing throughout the task, and this was irrespective of empathic skills (Fig. 2e). However, (3) high EPT participants instructed to act as followers were better able to pick up such regularities and therefore minimized their temporal lag from leaders, resulting in superior interpersonal coordination (Fig. 4). This result suggested that empathic skills might specifically boost the capacity to predict the timing of others’ actions.

### A bidirectional relationship between interpersonal coordination and social behavior

A growing number of studies have underlined a relationship between interpersonal coordination and social behavior^6,12^. In particular, converging evidence suggests that engaging in a collective rhythmic activity entailing interpersonal coordination increases social behavior. Measures of such pro-social effects include affiliation, trust, cooperation, closeness to others, perceived cohesion, and empathy^13,14,16–23,27^. These studies did not, however, examine the directionality of this relationship, and specifically the possibility that it is bidirectional, i.e. whether it is not only the case that interpersonal synchrony results in pro-social effects, but also that individuals who have stronger social personality traits are generally better at interpersonal coordination tasks. Our study addressed this latter question.

Here we specifically considered one social skill that is broadly regarded as a key ingredient in interpersonal processes: empathy^74,75^. We specifically considered a sub-scale of this multidimensional construct, i.e. empathic perspective taking^31,32^. This is the ability of humans to take the perspective of others in everyday life situations. We observed that individuals scoring relatively higher on this scale (high EPT), also own superior interpersonal synchronization skills. This result, taken together with previous research, is consistent with a bidirectional relationship between social skills and interpersonal synchronization. On the one hand, engaging in a coordination task is a sufficient condition to enhance social behavior (as previously shown by others). On the other hand, having a more social personality—high in empathic skills—might generally be linked to being relatively adept at interpersonal coordination.

### A mechanism promoting interpersonal coordination in empathic individuals

Our findings point to a strategy and mechanism that are potentially relevant to explaining how empathic individuals achieve superior interpersonal coordination. First, we observed that designated leaders, irrespective of their empathic score, increased the predictability of their actions by making their performance timing more regular (a strategy serving as a “coordination smoother”^62,64,72^). In other words, leaders attempted to assist followers by increasing the predictability of their performance, similarly so across high and low empathic participants. Remarkably, however, these similar cues were picked up differently by high and low empathic followers, with high EPT followers making better use of this information, and synchronizing more rapidly when their leaders’ timing was more predictable. In particular: when high empathic participants acted as followers, their temporal lag with respect to leaders was small (compared to low empathic participants). This suggests that more empathic participants were better at predicting the timing of their partners’ actions.

Such a predictive process could be implemented from a psychological and neurophysiological perspective via motor simulation, i.e. the brain’s capacity to represent another’s actions by means of an internal simulation^49,76–80^. This concept may constitute the neurophysiological instantiation of what is generally referred to as “taking the perspective of another”, which is the aspect of empathy we investigated here. In a previous study where we measured a neurophysiological index of motor simulation in participants engaged in a joint musical action task, we provided direct evidence showing that such a simulation process is indeed stronger in more empathic participants^43^. In a second study, we demonstrated that when motor simulation is temporally impaired by means of “virtual” brain lesions (induced via transcranial magnetic stimulation), participants’ ability to coordinate with a co-performer deteriorates, and more so in individuals having higher empathic skills^45^.

This emerging framework linking empathy with predictive motor simulation can potentially unite a number of observations and accounts associated with distinct research areas. In cognitive neuroscience, several studies have underlined the importance of motor simulation processes for both empathy^41–44,81^ and interpersonal coordination tasks^82–85^. Consistently with these empirical findings, a number of accounts from the fields of psychology and empirical musicology highlight the importance of empathic skills for coordination and collaboration^86–88^, and how these might emerge through enhanced linkages between perception and action^80,89,90^. Our work not only links these claims and observations from different fields, but also provides a potential mechanistic explanation of how empathy promotes interpersonal coordination. Specifically, our results indicate that more empathic individuals benefit from enhanced predictive skills when it comes to anticipating others’ behavior, and not just an augmented aptitude for cooperation. The notion of “aptitude for cooperation” refers to participants’ willingness to cooperate with their partners in order to maximize success in the task, as indexed indirectly by increased individual tempo stability across high and low EPT groups (i.e., a coordination smoother, which was observed in leaders independently of EPT). Our results instead favor an account where predictive skills provide empathic individuals with a behavioral advantage impacting social cognition on a broader scale.

### On the relationship between musical interaction and empathy

The relationship between empathy and music is a topic that received considerable recent interest^91–97^. In this area, several investigations have addressed a specific unidirectional relationship in which musical activities promote empathy. Music students, as compared to non-music students, have been shown to rate higher on empathy scales^98,99^. Consistently with this, other research has shown that children (without prior musical background) undergoing music programs enhance their empathic skills^14,100^. Notably, the study by Rabinowitch *et al*. (2013) showed that children engaged in musical group interactions developed higher emotional empathy^14^. These results are also supported by other investigations and claims suggesting that musical experience, entailing both sensory and motor musical activities, can promote empathy^91,101,102^.

None of these studies, however, examined whether individuals with higher empathic scores prior to musical training or related task experience are generally better in the context of a group musical interaction. One reason why this has not been explored could be that such a question is best addressed within musically untrained individuals, especially given that honing musical skills appears to be sufficient to enhance empathy. Here, this limitation was overcome by using the E-music box, a musical instrument that can be played by non-musicians and that can provide rich empirical information concerning the dynamical interaction within a pair^59^.

Our results indicate that more empathic individuals are predisposed to be more accurate at performing music with others, and that this benefit might originate from superior skill at temporal prediction. Taken together with previous findings^14,91,100,102,103^, this sheds light upon a bidirectional nature of the relationship between musical interaction and empathy. Such directionality suggests that the capacities to (1) coordinate with others and (2) empathize with others rely on a common mechanism whose functioning is modulated by experience in musical interaction as well as personality traits. As argued above, we claim that such a shared mechanism is motor simulation. We propose that empathic perspective taking might help ensemble musicians to coordinate with others by using information about a partner’s past action style (systematic timing variations) to predict their future action timing^104^. It should also be noted that this kind of benefit might be harder to observe in the context of simple isochronous tapping tasks (which are often employed) where there is little room for idiosyncratic timing (but mainly random variability), and therefore a critical lack of useful information about action style^80^. In music, by contrast, the challenge of producing a variable rhythm (i.e., different note durations) opens up the possibility for idiosyncratic timing patterns^105,106^ that, if someone is good at taking another’s perspective, are predictable. The present study hence suggests a plausible role of empathy in nonverbal communication, which can be conveniently—and with potential universal applicability—studied in the music domain, where there is ample room for individual variation.

### Coordination prediction and predictability as a function of information availability

A noteworthy advantage of musical paradigms, compared to standard finger tapping tasks, is that participants are engaged in a rich type of interaction, which can be formally described by a musical score^10^. Building on this, and on the fact that the density of notes was not uniform throughout the musical piece, we could compute an index that quantified how much information the two players could exchange throughout performance of the musical piece.

When we used this information to interpret our results, we made two noteworthy observations. Firstly, we observed that the interactive benefit of empathy and leadership began as soon as information about the leader’s timing started coming in at a relatively high rate (Fig. 3). This observation is reasonable in light of our interpretation of high EPT followers being better at predicting the leader’s action timing. However, the finding provides robust support for our interpretation to the extent that it highlights how the effect occurs selectively when participants begin having access to a sufficient amount of information. Thus, the temporal prediction process strongly depends on the amount of information that is available in a given time within the interpersonal coordination task.

Complementary to this first observation, we also observed that leaders’ enhanced predictability – presumably intended to help followers to synchronize – was mostly pronounced when information about the partner was less available (i.e. during segments of the musical piece that entailed lower note density, Fig. 3). This suggested that leaders took responsibility over keeping the correct tempo for the pair, particularly when less information exchange was available. This observation confirms previous studies suggesting that reducing the variability of one’s own actions might indeed be a powerful mechanism for achieving interpersonal coordination in the absence of continuous feedback about a co-actor’s actions^63,64^.

In sum, these observations strengthen the independence of predictive and cooperative behaviors. Specifically, EPT appears to boost sensorimotor processing in a manner that augments the prediction of others’ behavior in followers, but not the aptitude for cooperation in leaders.

## Conclusion

The present results provide direct evidence for a tight and bidirectional relationship between coordinated behavior and social cognition. Complementing previous research reporting pro-social effects of interpersonal coordination, we tested whether a social personality trait—empathic perspective taking (EPT)—is sufficient to enhance interpersonal coordination accuracy. We found that EPT promotes interpersonal coordination by specifically enhancing prediction accuracy for others’ behavior, while leaving the aptitude for cooperation unaffected. We suggest that such predictive capacity relies on a sensorimotor mechanism responsible for simulating others’ actions in an anticipatory manner, leading to behavioral advantages that may impact social cognition more generally.

## Data Availability

The data are available from the corresponding author upon request.
